# A Systematic Review of Maternal Serum Syndecan-1 and Preeclampsia

**DOI:** 10.7759/cureus.25794

**Published:** 2022-06-09

**Authors:** Kitty George, Prakar Poudel, Roopa Chalasani, Mastiyage R Goonathilake, Sara Waqar, Sheeba George, Wilford Jean-Baptiste, Amina Yusuf Ali, Bithaiah Inyang, Feeba Sam Koshy, Lubna Mohammed

**Affiliations:** 1 Research, California Institute of Behavioral Neurosciences & Psychology, Fairfield, USA; 2 Pediatrics, California Institute of Behavioral Neurosciences & Psychology, Fairfield, USA

**Keywords:** eph complex, cd-138, syndecan-1, sdc-1, preeclampsia

## Abstract

Exploration of novel biomarkers has been gaining popularity in preeclampsia, which is currently being diagnosed based on clinical criteria alone. Soluble syndecan-1, released from one of the proteoglycans associated with the syncytiotrophoblastic layer of the placenta, is affected in patients with abnormal placentation. This article is the first systematic literature review that evaluates the relationship between the antepartum serum levels of the syndecan-1 and preeclampsia. Eight studies were selected after screening and quality appraisal, and data were analyzed. The serum concentration of syndecan-1 was found to correlate positively with the gestational age in all pregnancies and negatively with the systolic blood pressure in patients with preeclampsia. Extremely low levels of soluble syndecan-1 may be helpful as a predictor for the development of preeclampsia during gestation.

## Introduction and background

It is estimated that over half a million women die every year due to pregnancy-related complications worldwide [1]. Preeclampsia, one of the most dreaded diseases of pregnancy, imparts substantial morbidity and mortality to pregnant women and their fetuses alike. Classically defined as new-onset hypertension with signs of widespread maternal end-organ damage, evidenced by proteinuria occurring after 20 weeks of gestation, preeclampsia critically affects the fetoplacental system, leading to debilitating fetal and maternal outcomes [2]. In addition to being a part of the broad spectrum of hypertensive disorders of pregnancy [3], preeclampsia alone complicates 2-8% of all pregnancies affecting primigravida up to two times more than the multiparous women [4]. While the exact etiology of preeclampsia is still debatable, various clinicopathological studies have demonstrated that the central culprit is the placental vasculature [5].

Being a disease of the placenta, the only definite management of preeclampsia is the termination of pregnancy [6]. Therefore, several interventions have been attempted to prevent preeclampsia from causing its detrimental effects on fetomaternal health. Of these, prophylactic treatment with low-dose aspirin shows promising results along with heparin and calcium [7-10]. However, the problem lies with the early detection of preeclampsia, which is still clinically diagnosed after 20 weeks of gestation. Novel biomarkers like placental-derived exosomes [11], soluble endoglin, and soluble FMS-like tyrosine kinase [12] provide excellent clinical utility for recognizing those women at high risk for the development of preeclampsia by identifying the potential candidates for preventive strategies. These biomarkers, either in isolation or in combination with other factors [13], can assist in the early diagnosis and future management by developing integrated clinical risk models [14].

Syndecans, including the syndecan-1 (SDC-1) primarily seen on the epithelial cells, constitute a family of transmembrane heparan sulfate proteoglycans consisting of four members, differing from each other based on their highly variable extracellular domains [15]. During pregnancy, SDC-1 seemingly affects cell signaling and intercellular interaction at the trophoblast-decidual interface [16]. While usually present on the cell surface, the ectodomains of these proteoglycans can be shed from the cell surface, generating soluble molecules that can inhibit interactions at the cell surface [17]. The process of converting the cell-bound SDC-1 into an active soluble ligand form by sheddases is key to many inflammatory processes [18-20].

Even though soluble SDC-1 is already being explored as a valid biomarker in many conditions like inflammatory bowel disease [21], organ dysfunction, endothelial injury [22], and cancer [23], the association between SDC-1 and preeclampsia has not been sufficiently reviewed. As of April 2022, there is no systematic review or meta-analysis on this subject, despite the adequate number of published analytical studies. Therefore, this review aims to shed light on this area by collecting and critically analyzing the existing body of evidence to identify, collect, and help investigate the association between preeclampsia and maternal serum SDC-1 levels during pregnancy.

## Review

Methods

This systematic review was conducted in accordance with the updated 2020 Preferred Reporting Items for Systematic Reviews and Meta-Analyses (PRISMA) guidelines [24].

Eligibility Criteria

All analytical observational studies in the English language exploring the relationship between soluble SDC-1 levels in pregnant patients and preeclampsia were included in the review. Animal studies, opinion articles, editorials, case reports or series, cases with twin gestation, and studies measuring only the postpartum levels of serum SDC-1 were excluded from the study.

Search Strategy

A thorough article search was done using the databases of PubMed, PubMed Central (PMC), and Medline, and further reports were sought using DeepDyve, Google Scholar, and Bielefeld Academic Search Engine (BASE) using the keywords mentioned in Table 1, along with the Boolean operators as required.

**Table 1:** Databases and keywords used PMC: PubMed Central; BASE: Bielefeld Academic Search Engine.

Database/website	Keywords used	Filters/limits	Date of the last search
PubMed	("preeclampsia" OR "pre-eclampsia" OR "pre eclampsia" OR "pregnancy toxemia" OR "EPH complex" OR "Edema-Proteinuria-Hypertension Gestosis") AND ("syndecan 1" OR "syndecan-1" OR "sdc 1" OR "sdc-1" OR "CD 138" OR "CD-138")	N/A	4/24/2022
PMC	"Pre-Eclampsia"[Mesh] AND "Syndecan-1"[Mesh]	N/A	4/24/2022
Medline	("preeclampsia" OR "pre-eclampsia" OR "pre eclampsia" OR "pregnancy toxemia" OR "EPH complex" OR "Edema-Proteinuria-Hypertension Gestosis") AND ("syndecan 1" OR "syndecan-1" OR "sdc 1" OR "sdc-1" OR "CD 138" OR "CD-138")	N/A	4/24/2022
DeepDyve	preeclampsia + (soluble OR serum OR circulatory OR circulating) "syndecan-1"	N/A	4/24/2022
Google Scholar	preeclampsia soluble OR serum OR circulatory OR circulating "syndecan-1"	Switched off keyword search within the citations list	4/24/2022
BASE	preeclampsia syndecan-1	N/A	4/24/2022

Study Selection and Data Collection

All reports acquired from the National Library of Medicine (NLM), PMC, and Medline were exported to EndNote Online (Clarivate, London, UK) and deduplicated prior to manual screening based on title and abstract. The reports obtained from DeepDyve, Google Scholar, and BASE were directly screened after reading the titles. Each retrieved article was read completely from abstract to conclusion to ensure that the inclusion criterion was satisfied and whether any of the exclusion factors were met. Attempts were made to obtain additional articles by doing a citation search of the already included studies. Data collection was done by two researchers (KG and PP) independently, and the variables included various study characteristics along with serum SDC-1 levels. Qualitative variables were mentioned as percentages. The quantitative variables were expressed as mean ± standard deviation (SD) for normal distributions and median with interquartile range (IQR) for variables with a skewed distribution.

Bias Risk and Study Quality Assessment

Assessment of the risk of bias and quality of the included studies was independently completed by the two researchers (KG and PP). The Newcastle-Ottawa Scale [25] was used for observational analytical studies, including case-control and cohort studies. Cross-sectional studies were assessed using the Appraisal Tool for Cross-Sectional Studies (AXIS) [26]. When there was a disagreement between the authors, a consensus was sought. Those reports with quality scores of less than 70% were omitted from further analysis and syntheses.

Results

**Figure 1:**
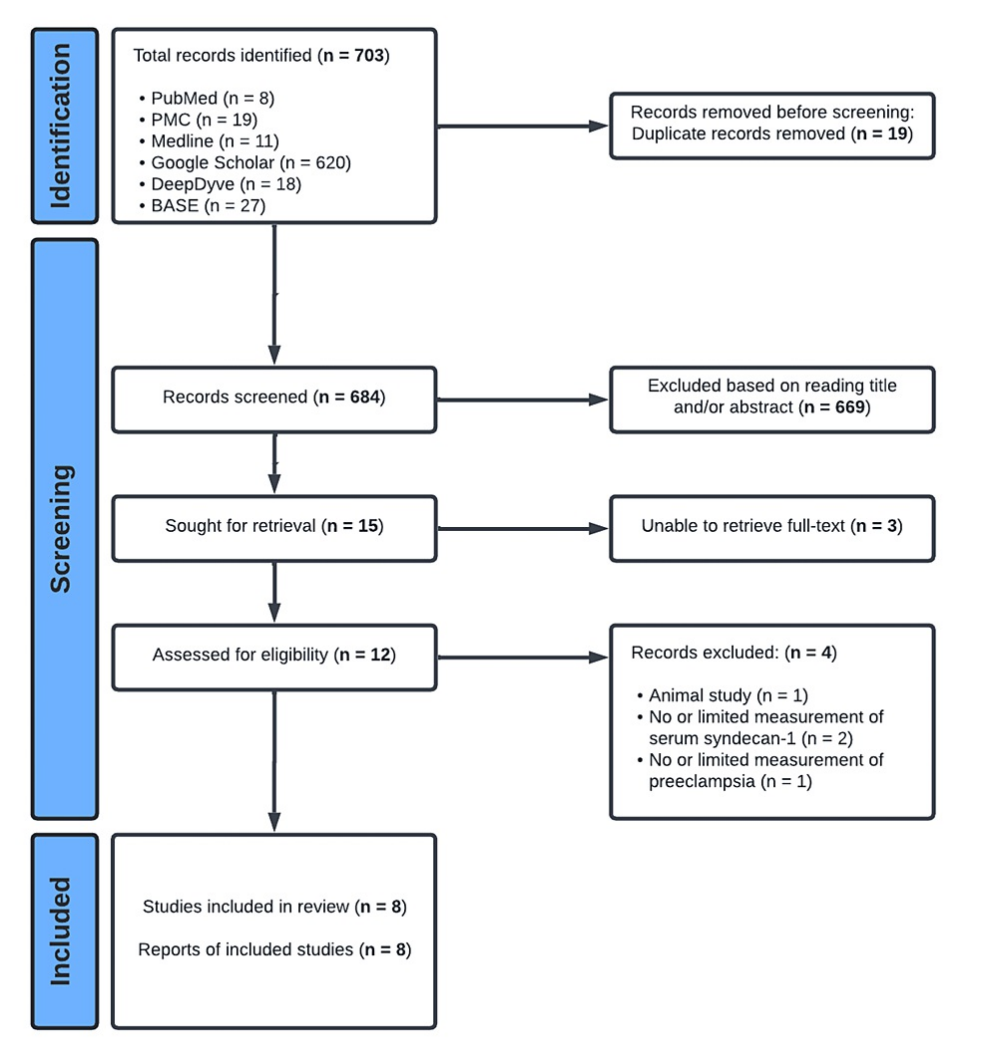
Flow diagram of study selection PMC: PubMed Central; BASE: Bielefeld Academic Search Engine. Created using Lucidchart (Lucid Software Inc., South Jordan, UT).

The search strategy for the identification of relevant reports, as elaborated in the methods section, yielded 703 records, which were reduced to 684 after using the deduplication function in the EndNote Online, all of which were screened by title and/or abstract reading. Three articles were not included because the full text was not retrievable. Out of the 12 records that passed screening and retrieval, eight were included in the review because four articles met the exclusion criteria previously set. Two studies [27,28] that appeared to have met the eligibility criteria were not included because they contained only limited information regarding the measurement of serum SDC-1 levels. Figure 1 illustrates the PRISMA flow diagram demonstrating the study identification, selection, and inclusion process used in the current review.

Bias Risk

Table 2 shows the assessment done for the risk of bias estimation in the studies selected for review.

**Table 2:** Assessment of the risk of bias NCO: Newcastle-Ottawa; AXIS: Appraisal Tool for Cross-Sectional Studies; SDC-1: syndecan-1; BMI: body mass index.

Study source	Study type	Risk of bias tool used	Score (percentage)	Remarks
Lahsinoui et al. (2021) [29]	Cross-sectional	AXIS	72.5%	Hospital controls were used. Four patients from the case group and one from the control group lacked information regarding serum SDC-1 levels in the antepartum period.
Greeley et al. (2020) [30]	Retrospective cohort and case-control	NCO	77.8%	Only the case-control part was included for purpose of this review. There was a significant difference between the cases and controls with smoking history (p = 0.035).
Kuessel et al. (2019) [31]	Nested case-control	NCO	77.8%	There was a significant difference in parity between the additionally recruited cases and controls (p = 0.004).
Kornacki et al. (2019) [32]	Cross-sectional	AXIS	70.0%	There was a significant difference in BMI between the cases and controls (p < 0.05).
Webster et al. (2018) [33]	Nested cohort/cross-sectional	AXIS	82.5%	There was a significant difference in BMI and co-existence of diabetes between the cases and controls (p < 0.05).
Davutoğlu et al. (2017) [34]	Cross-sectional case-control	AXIS	80.0%	There was a significant difference in BMI and timing of SDC-1 measurement between the groups.
Gandley et al. (2016) [35]	Nested case-control	NCO	77.8%	No adjustment for parity was considered.
Szabo et al. (2013) [36]	Cross-sectional	AXIS	72.5%	There was a significant difference in parity between the groups.

Study Characteristics

All eight studies selected for review were comparative observational studies involving 1081 participants. SDC-1 levels measured during the gestational period were recorded. The cost was not used in any studies as an outcome measure. Table 3 shows the characteristics of the final included studies.

**Table 3:** Characteristics of included studies BMI: body mass index; IQR: interquartile range; HELLP: hemolysis, elevated liver enzymes, and low protein.

Study source	Study title	Study population	Maternal age (years)	BMI	Nulliparity (%)	Smoking (%)	Gestation at delivery (weeks)	Neonatal birth weight (grams)
Lahsinoui et al. (2021) [29]	Soluble Syndecan-1 and Glycosaminoglycans in Preeclamptic and Normotensive Pregnancies	125 participants						
		65 preeclamptic cases	31 ± 5	27 ± 6	57%	-	33+5/7 ± 3+6/7	1914 ± 832
		60 normotensive controls	29 ± 6	25 ± 7	58%	-	34+4/7 ± 3+6/7	2292 ± 876
Greeley et al. (2020) [30]	Evaluation of Syndecan-1 as a Novel Biomarker for Adverse Pregnancy Outcomes	357 participants						
		119 cases of women with adverse pregnancy outcomes of which 101 had preeclampsia	32 (IQR: 29-36)	31 (IQR: 27.1-35.5)	36.1%	2.5%	34+6/7 (IQR: 32+2/7 - 36+1/7)	1920 (IQR: 1460-2480)
		238 controls	32 (IQR: 29-35)	31.2 (IQR: 27.5-35.3)	28.2%	0%	39+4/7 (IQR: 39+0/7 - 40+2/7)	3420 (IQR: 3192-3710)
Kuessel et al. (2019) [31]	Dynamics of soluble syndecan-1 in maternal serum during and after pregnancies complicated by preeclampsia: a nested case control study	153 participants						
		12 cases as the part of a cohort	32.3 ± 5.2	26.8 ± 4.0	25%	17%	34.8 ± 4.6	2030.8 ± 820.4
		47 symptomatic cases of preeclampsia additionally recruited	32.6 ± 6.8	25.5 ± 4.8	67%	9%	32.4 ± 4.2	1696.8 ± 978.7
		95 controls	30.2 ± 5.4	24.7 ± 5.2	42%	21%	39.5 ± 1.3	3412.7 ± 451.9
Kornacki et al. (2019) [32]	Levels of Syndecan-1 and Hyaluronan in Early- and Late-Onset Preeclampsia	60 participants						
		20 cases with early-onset preeclampsia (diagnosed before 34 weeks of gestation)	28 (IQR: 25 - 41)	26.4 (IQR: 21.8-40.6)	38%	-	32 (IQR: 26-37)	1319 ± 568
		20 cases with late-onset preeclampsia (diagnosed at ≥34 weeks of gestation)	31 (IQR: 19-40)	34.3 (IQR: 22.7-53.2)	67%	-	37 (IQR: 34-39)	2636 ± 683
		20 controls with normal pregnancies	32 (IQR: 27-36)	25 (IQR: 22-29)	56%	-	39 (IQR: 37-41)	3437 ± 554
Webster et al. (2018) [33]	Chronic Hypertension in Pregnancy: Impact of Ethnicity and Superimposed Preeclampsia on Placental, Endothelial, and Renal biomarkers	117 participants						
		25 patients with chronic hypertension with preeclampsia	33 ± 6	31 ± 5.8	28%	0%	34.7 (IQR: 30.3-37.9)	2270 (IQR: 1320-2840)
		92 patients with chronic hypertension without superimposed preeclampsia	36 ± 5	31 ± 6.3	16	1.1%	38.3 (IQR: 37-39.3)	3020 (IQR: 2690-3440)
Davutoğlu et al. (2017) [34]	Evaluation of Maternal Serum Hypoxia Inducible Factor-1a, Progranulin and Syndecan-1 levels in Pregnancies with Early- and Late-Onset Preeclampsia	80 participants						
		27 cases with early-onset (<34 weeks) preeclampsia	31.6 ± 5.5	31.2 ± 4.8	-	0%	30.85 ± 3.34	1386 ± 756
		27 cases with late-onset (≥34 weeks) preeclampsia	29.4 ± 5.2	31.9 ± 4.6	-	0%	37.37 ± 1.62	2825 ± 670
		26 controls with normal pregnancies	28.8 ± 5.3	27.9 ± 1.6	-	0%	38.46 ± 1.52	3334 ± 467
Gandley et al. (2016) [35]	Low Soluble Syndecan-1 Precedes Preeclampsia	44 participants						
		19 cases with preeclampsia	28 (IQR: 17-36)	27 (IQR: 21 - 36)	-	5%	33 (28-40)	1825 (561-3400)
		25 cases with uncomplicated pregnancies	28 (IQR: 20-38)	27 (IQR: 20-50)	-	8%	39 (24-42)	3005 (525-3889)
Szabo et al. (2013) [36]	Changes of Placental Syndecan-1 Expression in Preeclampsia and HELLP Syndrome	81 participants						
		49 cases with preeclampsia	23 (IQR: 19.2-28.5)	26.8 (IQR: 23-32.6)	51%	-	34.6 (IQR: 30.1-37.7)	1,825 (IQR: 1,070-2,635)
		32 controls	25.5 (IQR: 20-31)	26.4 (IQR: 21.7-32.8)	25%	-	36.8 (IQR: 29.9-38.8)	3,073 (IQR: 1,258-3,268)

Syndecan-1 Concentrations During Pregnancy

The results extracted from the selected studies are shown in Table 4.

**Table 4:** Serum syndecan-1 levels during gestation SDC-1: syndecan-1; IQR: interquartile range; HELLP: hemolysis, elevated liver enzymes, and low protein; MoM: multiples of the median.

Study source	Groups	Antepartum SDC-1 levels (ng/mL)	Remarks	Conclusions related to serum SDC-1
Lahsinoui et al. (2021) [29]	Cases with preeclampsia with HELLP	644 (IQR: 286-919)	Levels were measured prior to delivery and three months postpartum.	Soluble SDC-1 plasma levels in preeclamptic and normotensive women were similar. However, in preeclamptic women, soluble SDC-1 level was inversely correlated with systolic blood pressure (r = 0.29, p = 0.02).
	Cases with preeclampsia without HELLP	447 (IQR: 267-734)		
	Total cases with preeclampsia	553 (IQR: 309-805)		
	Normotensive controls	551(IQR: 307-920)		
Greeley et al. (2020) [30]	Cases of adverse pregnancy outcomes (101 with preeclampsia)	Values were not mentioned in the case-control sub-study	There was no significant difference (p = 0.22) in maternal SDC-1 values measured from first-trimester aneuploidy serum screening samples.	Serum SDC-1 levels significantly increase during gestational weeks 11 to 13. Extremely low first trimester SDC-1 levels can be associated with adverse pregnancy outcomes (MoM ≤ 0.5, OR = 3, p = 0.003).
	Controls			
Kuessel et al. (2019) [31]	Cases as the part of a cohort	7.83 (IQR: 5.82-11.71), 12.42 (IQR: 9.30-17.08), 22.49 (IQR: 17.38-24.97), 20.58 (IQR: 14.45-27.61), 29.30 (IQR: 27.35-29.87)	For levels measured at 20, 25, 30, 35, and 38 weeks.	Lower levels of SDC-1 may be useful in predicting the development of preeclampsia.
	Additionally recruited cases	13.79 (IQR: 11.08-14.67), 19.32 (IQR: 15.90-23.20), 31.05 (IQR: 25.79-39.65), 34.67 (IQR: 27.49-41.22)	For levels measured at 25, 30, 35, and 38 weeks.	
	Controls	10.95 (IQR: 8.15-15.79), 17.90 (IQR: 13.29-26.12), 26.24 (IQR: 19.43-36.69), 29.54 (IQR: 22.06-41.35), 32.79 (IQR: 23.90-45.43)	For levels measured at 20, 25, 30, 35, and 38 weeks.	
Kornacki et al. (2019) [32]	Cases with early-onset (<34 weeks) preeclampsia	6.17 ± 2.2	Levels were measured on the day of diagnosis of preeclampsia (usually in the late second or third trimester).	The significance of a lower concentration of SDC-1 in patients with preeclampsia than in normotensive pregnant women needs further evaluation.
	Cases with late-onset (≥34 weeks) preeclampsia	6.42 ± 2.2		
	Total cases with preeclampsia	6.29 ± 2.18		
	Controls with normal pregnancy	11 ± 2.62		
Webster et al. (2018) [33]	Chronic hypertension without preeclampsia	Values were not mentioned within the report	SDC-1 concentrations increased significantly across gestation in all groups (p < 0.0001).	There was no statistically significant difference in SDC-1 concentrations either in women with chronic hypertension who did or did not develop superimposed preeclampsia (p = 0.62).
	Chronic hypertension without superimposed preeclampsia			
Davutoğlu et al. (2017) [34]	Cases with early-onset (<34 weeks) preeclampsia	22.6 (IQR: 19.8-24.9)	Blood samples were taken at the time of admission (ranging from 25 to 40 weeks). Significant decreases in serum SDC-1 levels were noted in preeclampsia compared to the control group and were significantly more decreased in the early-onset group. However, there was a significant difference between groups in the timing of SDC-1 measurement.	SDC-1 can be used as a marker of placentation problems and seems to be predominantly decreased in patients with early-onset preeclampsia.
	Cases with late-onset (≥34 weeks) preeclampsia	29.5 (IQR: 24.5-31.5)		
	Total cases with preeclampsia	25.9 (IQR: 20.7-29.5)		
	Controls with normal pregnancies	30.5 (IQR: 21.1-133.8)		
Gandley et al. (2016) [35]	9 cases with preeclampsia at mid-pregnancy (18-24 weeks)	174 (IQR: 48-353)	Levels were significantly decreased in cases with preeclampsia when compared to the controls and gestational hypertension.	Maternal plasma levels of soluble SDC-1 rise approximately 50-fold with gestation and revert postpartum. Women who later develop preeclampsia have lower levels of soluble SDC-1 in maternal plasma at the gestational age of 20 weeks (before clinical disease onset) compared to women with uncomplicated pregnancy or gestational hypertension.
	9 cases with gestational hypertension at mid-pregnancy (18-24 weeks)	242 (IQR: 111-1187)		
	19 controls with uncomplicated pregnancy (18-24 weeks)	272 (IQR: 78-1463)		
	17 cases with preeclampsia in the third trimester	281 (IQR: 101-2237)	Circulating soluble SDC-1 was ~2.5-fold lower in women with preeclampsia. There was a significant difference between the groups (p < 0.01).	
	17 normotensive controls before delivery	705 (IQR: 243-2861)		
Szabo et al. (2013) [36]	Cases with preeclampsia	673 (IQR: 459-1161)	Maternal serum SDC-1 concentration was lower in preeclampsia, even after adjusting for gestational age (p = 0.03). The levels were negatively correlated with mean arterial pressure (R^2^ = 0.08, p = 0.012) and positively correlated with gestational age (R^2^ = 0.22, p = 1×10^−5^) and birth weight (R^2^ = 0.25, p = 1.7×10^−6^).	Trophoblastic SDC-1 release is decreased in preeclampsia and HELLP syndrome.
	Controls	1158 (IQR: 622-1480)		

Discussion

Preeclampsia, defined as new-onset hypertension and proteinuria or other end-organ damage occurring after 20 weeks of pregnancy, is characterized by abnormal placentation caused by inadequate trophoblast invasion and defective spiral artery remodeling, which leads to ischemia and oxidative stress at the maternal-fetal interface. Subsequently, the ensuing release of cytotoxic substances and antiangiogenic factors into the maternal circulation result in widespread endothelial and end-organ damage [37], including the kidney, liver, and brain. The fetus is also affected by the placental insufficiency arising from an abnormal interaction between the fetal trophoblast and maternal decidua, which can lead to growth restriction, preterm labor, and stillbirth [38].

**Figure 2:**
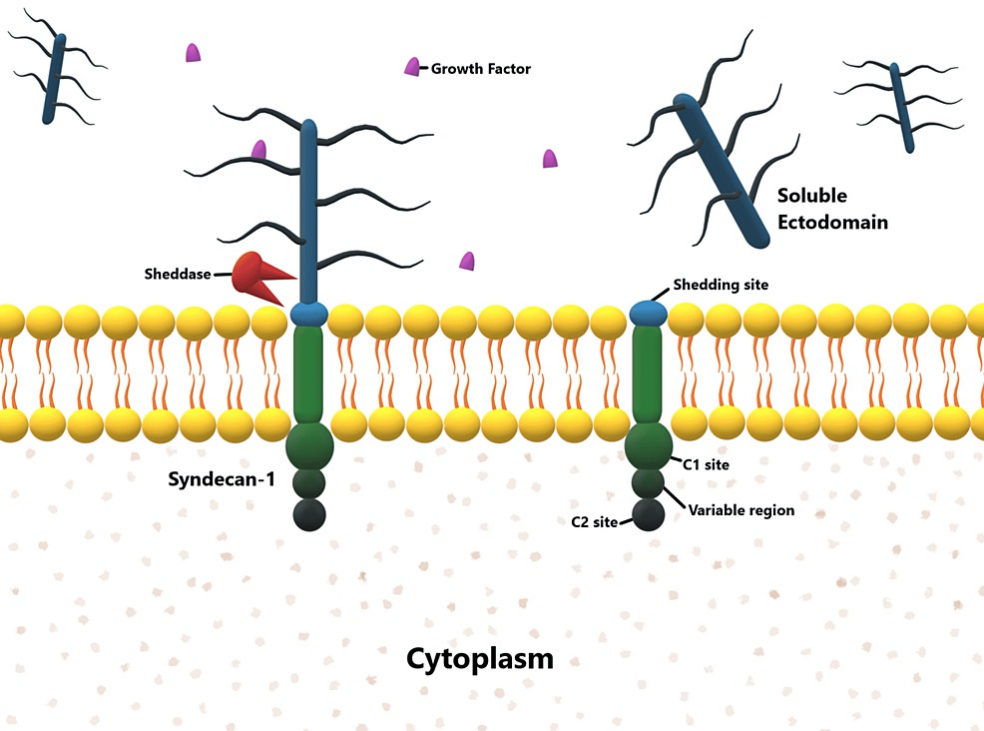
Shedding of transmembrane syndecan-1 and formation of soluble syndecan-1 Created using Microsoft Paint 3D (Microsoft Corporation, Redmond, WA) by George Michael.

The two-stage theory proposes that placental disease precedes maternal endothelial dysfunction [39]. Therefore, molecules released from the placenta (e.g. syndecans) are likely to show the first changes during the development of preeclampsia. Syndecans are known to bind to various growth factors and cytokines and consequently regulate signal transductions [40]. They thus play an important role in modulating cellular signaling in embryonic development, tumorigenesis, and angiogenesis [41]. SDC-1, the most abundant member of the syndecan family of proteoglycans, is strongly localized to the syncytiotrophoblast membrane of the chorionic villi. By forming a complex with vascular endothelial growth factor (VEGF) receptor, SDC-1 regulates cell signaling, thereby modulating VEGF-induced motility and migration of endothelial cells [42]. Proteolytic shedding of the ectodomain of SDC-1 occurs continuously, and because they are concentrated in the apical membrane of the chorionic villi, SDC-1 remnants may be seen in the plasma as soluble SDC-1. This concept is illustrated in Figure 2.

The placenta acts as a major source of SDC-1 during pregnancy. Therefore, serum SDC-1 levels rise throughout pregnancy, with the greatest increase between 20 and 30 weeks [43]. After delivery, the concentrations fall rapidly, consequent to the loss of the placenta. SDC-1 transport to the cell surface is dependent on intact cytoskeletal proteins like actin, which may be disrupted in preeclampsia [44] and results in increased retention within the cells. Consequently, the placental expression of SDC-1 changes, which precedes the development of clinical features in preeclampsia [45]. The alteration in the cell surface expression with preeclampsia manifests as a decrease in soluble SDC-1 levels, measured as the serum SDC-1.

The differences between serum SDC-1 levels start before the onset of disease in preeclampsia [35] and remain evident even after disease onset, especially in those with fetal growth restriction. Szabo et al. have postulated that altered transport of the placental SDC-1 may lead to inflammatory changes in the trophoblast. Since soluble SDC-1 retains its property to bind cytokines, decreased shedding can promote or exaggerate maternal systemic inflammation related to preeclampsia. Additionally, because of its ability to bind various growth factors like fibroblast growth factor 2, hepatocyte growth factor, VEGF, and transforming growth factor-beta, decreased levels of SDC-1 may also play a central role in the antiangiogenic state of preeclampsia [36].

Findings from the included reports, all of which were comparative analytical types, were heterogeneous. Most studies excluded subjects with multiple pregnancies, systemic inflammation, or diabetes, which could be potential confounders for preeclampsia and SDC-1. The control groups from all the studies were normotensive, except that of Webster et al. (2018) [33], which used a control group with chronic hypertension. Studies from Lahsinoui et al. (2021) [29], Greeley et al. (2020) [30], and Webster et al. (2018) [33] did not show a statistically significant association between preeclampsia and serum SDC-1, while those from Kuessel et al. (2019) [31], Kornacki et al. (2019) [32], Davutoğlu et al. (2017) [34], Gandley et al. (2016) [35], and Szabo et al. (2013) [36] showed significantly lower SDC-1 levels with preeclampsia when compared to normal pregnancies. However, Greeley et al. (2020) [30] also noted that extremely low levels of SDC-1 during the first trimester are associated with an increased risk of preeclampsia. They used multiples of the median (MoM) to determine the deviation of serum SDC-1 from the median value. MoM = SDC-1 concentration ÷ median SDC-1 for the gestational age determined from the retrospective cohort. It was estimated that MoM less than or equal to is associated with an odds ratio of 3.0 (p = 0.003) in preeclampsia when compared to normal pregnancies.

One interesting finding from the study by Gandley et al. (2016) [35] was the absence of a significant difference between serum SDC-1 levels of gestational hypertensive patients and normotensive pregnancies. This implies that SDC-1 levels are intricately linked to the placental pathology, which makes preeclampsia distinct from gestational hypertension. Additionally, multiple studies have found a negative correlation between systolic blood pressure and SDC-1 levels in preeclampsia. This would seem intuitive as a significant placental abnormality, and therefore, lower serum SDC-1 levels are expected with higher blood pressure. More investigation is required to explore this aspect, preferably in the form of prospective cohort studies.

We believe that measuring SDC-1 at specific times like during diagnosis, admission, before delivery, or during delivery and then using the levels to compare preeclamptic and normal pregnancies does not give meaningful information because SDC-1 appears to be a function of gestational age. It is always better to compare SDC-1 levels that have been adjusted for gestational age. The usefulness of measuring SDC-1 concentrations for predicting the development of preeclampsia by analyzing receiver operating characteristic (ROC) curves was described by Kuessel et al. (2019) [31]. However, the predictive power was different between various groups of the study, and a significant predictive power was noted between 16 and 36 weeks.

It is interesting to note that serum SDC-1 level, even though increasing throughout pregnancy, is relatively decreased when placental damage occurs. This trend is opposite to that of other proteoglycan and glycocalyx components of the placenta, which increases with an injury like in preeclampsia. The inverse relationship between SDC-1 levels and blood pressure in patients with preeclampsia needs to be explored further. Future prospective studies may be conducted by comparing SDC-1 levels in patients with preeclampsia but should be adjusted for gestational age and/or fetal weight. We are hopeful that this systematic review inspires further research into using biomarkers because of the potential to diagnose preeclampsia far before the clinical syndrome develops, giving a window of opportunity for the administration of prophylactic therapy.

Limitations

While the number and quality of studies included were adequate, there was a paucity of prospective studies that could be useful in ascertaining the genuine relationship between serum SDC-1 and preeclampsia. The conflicting results from the studies included made it challenging to form a unified conclusion from this review. Furthermore, there was only limited information regarding the timing of serum SDC-1 measurement in some of the selected reports. Since serum SDC-1 concentration is known to change with the gestational age, this resulted in the inability to compare the values from different studies directly. Other studies attempted to compare the SDC-1 levels in different groups without adjusting for gestational age, potentially distorting their conclusions.

## Conclusions

This article pioneers in systematically reviewing the relationship of antepartum levels of the soluble ectodomain portion of SDC-1 in the serum of pregnant women with preeclampsia. SDC-1 likely plays a crucial role in the pathogenesis of preeclampsia and increases significantly throughout gestation. Therefore, when comparing the serum concentrations of SDC-1 between the different patient groups, adjusting for gestational age can be helpful in identifying a true association. Although conflicting reports are available regarding the significance of the association, extremely low levels of serum SDC-1 can reliably be considered a predictor of preeclampsia. The inverse correlation between SDC-1 levels and blood pressure in patients with preeclampsia needs to be explored further.
